# Clinical efficacy and pharmacokinetic study of antituberculosis treatment in patients with end-stage renal disease undergoing hemodialysis

**DOI:** 10.2478/abm-2026-0021

**Published:** 2026-06-30

**Authors:** Wei Song, Zhao Yang, Yuqian Zhao, Bing Liu, Musong Li, Wei Du, Ning Niu, Jianru Jia

**Affiliations:** Blood Purification Unit, Baoding People’s Hospital, Baoding City 071000, Hebei, China; Quality Control Department, Baoding People’s Hospital, Baoding City 071000, Hebei, China; Infection and Severe Disease Department, Baoding People’s Hospital, Baoding City 071000, Hebei, China; Baoding People’s Hospital, Baoding City 071000, Hebei, China; Department of Hepatology, Integrated Traditional Chinese and Western Medicine, Baoding People’s Hospital, Baoding City 071000, Hebei, China

**Keywords:** adjustment of dose, end-stage renal disease, hemodialysis, pharmacokinetics, tuberculosis

## Abstract

**Background:**

Patients with end-stage renal disease (ESRD) undergoing hemodialysis exhibit a 10–25 times greater risk of tuberculosis (TB), underscoring the urgent need for optimized treatment strategies to improve prognosis.

**Objective:**

The present study evaluates the efficacy and pharmacokinetics of personalized anti-TB dosing in hemodialysis patients with ESRD and TB, to guide treatment optimization.

**Methods:**

This retrospective study included 90 hemodialysis patients with ESRD and TB (2020 – 2024). The control group (n = 45) received conventional anti-TB dosing; the study group (n = 45) received individualized dosing/timing. All received HRZE (isoniazid, rifampicin, pyrazinamide, ethambutol) and high-flux hemodialysis or hemodiafiltration. Efficacy endpoints included sputum conversion (4, 8, and 12 weeks), imaging at 12 weeks, and clinical effectiveness. Blood samples were collected before/during/after/dialysis intervals to calculate C_max_, C_min_, AUC_0–24_, CL, CLDAA, ER, t^½^, and adverse reactions.

**Result:**

No significant baseline differences existed between groups (*P* > 0.05). The study group showed higher sputum conversion rates, 12-week radiographic improvement, and overall effectiveness (*P* < 0.05). Pharmacokinetic (PK) analysis revealed elevated C_max_, C_min_, AUC_0–24_ for all anti-TB drugs (*P* < 0.05). Rifampicin clearance was unchanged (*P* > 0.05), while others decreased (*P* < 0.05). Half-life shortened on dialysis days (*P* < 0.01). Adverse reactions were markedly reduced in the study group (*P* < 0.01).

**Conclusions:**

For hemodialysis patients with ESRD and TB, tailoring anti-TB drug regimens based on PK profiles can enhance efficacy and minimize adverse effects, offering a safe and personalized treatment pathway.

End-stage renal disease (ESRD), representing the terminal phase of progressive chronic kidney conditions, is increasingly prevalent worldwide. The rising global ESRD population imposes substantial medical and economic burdens on both societies and families [1, 2]. In China, factors such as population aging and growing rates of diabetes and hypertension have further contributed to a marked increase in ESRD cases [3, 4]. Hemodialysis, as a major renal replacement therapy, maintains the survival of patients, but it cannot completely restore normal physiological functions, and long-term dialysis will bring a series of complications [5, 6].

Tuberculosis (TB), caused by *Mycobacterium tuberculosis*, remains a critical global public health issue. The World Health Organization reports millions of new TB cases annually, with high associated morbidity and mortality. While pulmonary TB is most common, extrapulmonary cases also constitute a significant proportion. Recent increases in population mobility, the size of immunocompromised populations, and the emergence of drug-resistant strains have further complicated the TB control efforts. ESRD patients are particularly vulnerable due to their compromised immunity and frequent exposure to healthcare settings during dialysis [7, 8]. Studies indicate that the risk of TB in dialysis patients is 6.0–52.5 times higher than that in the non-chronic kidney disease (CKD) population, with prevalence rates of 3.3% in hemodialysis and 1.2% in peritoneal dialysis [9]. Furthermore, research reports indicate that in the general TB population, the sputum culture conversion rate at 2 months is approximately 60%–70%, while in hosts with weakened immune functions, such as ESRD patients, this proportion is often even lower [10]. Coexisting TB and ESRD not only complicates clinical management but also significantly elevates mortality and adversely affects patient survival and prognosis. Hence, developing effective treatment strategies for this population is imperative yet challenging.

Anti-TB drugs can exacerbate renal load in ESRD patients, with rifampicin frequently inducing acute kidney injury (AKI) via immune-mediated allergy or direct nephrotoxicity [11]. The increase of uric acid caused by pyrazinamide and ethambutol can cause renal damage. Uric acid crystals deposit in the renal tubules, blocking the renal tubules and affecting the normal excretion function of the kidney. In addition, isoniazid, streptomycin, and rifapentine (Rt) have also been reported to cause renal damage [12]. This makes it necessary to carefully weigh the relationship between drug efficacy and renal toxicity when selecting anti-TB drugs for ESRD patients. The clinical manifestations of ESRD patients with TB are often atypical, which overlap with the symptoms of uremia itself, increasing the difficulty of diagnosis [13]. TB in CKD patients is not confined to the lungs but spreads to multiple parts of the body [7]. Its clinical manifestations are mostly systemic symptoms, such as fever, fatigue, cough or hemoptysis, loss of appetite, weight loss, etc. These symptoms are similar to the common symptoms of patients with chronic renal failure, and it is difficult to accurately determine whether it is complicated by TB infection based on symptoms. This requires clinicians to improve the vigilance of ESRD patients with TB and comprehensively use a variety of diagnostic methods to achieve early and accurate diagnosis [14, 15].

Many factors, such as decreased glomerular filtration rate (GFR), hypoproteinemia, use of immunosuppressive agents, comorbidities, and dialysis, lead to significant changes in the pharmacokinetics of antituberculosis drugs in ESRD patients undergoing hemodialysis. Isoniazid and ethambutol are highly dialyzable and can remove approximately 50%–90% of their doses during a single hemodialysis session, which means that large quantities of the drug are removed from the body during dialysis, resulting in significant decreases in blood drug concentrations, and dose supplementation or adjustment after dialysis is often recommended to maintain the effective therapeutic concentrations needed. Pyrazinamide, which is moderately dialyzable with 30%–50% clearance, still needs to be administered after dialysis to ensure efficacy. However, rifampicin is a low-dialyzable drug due to its high protein binding and large molecular size, and the clearance rate is usually <20%. Therefore, dose adjustment of rifampicin may be less critical than other highly dialyzable drugs. These changes complicate the antituberculosis treatment of such patients, requiring the development of individualized dosing regimens to ensure drug efficacy and reduce toxic side effects [16, 17]. Nevertheless, pharmacokinetic (PK) research on anti-TB drugs in ESRD patients remains inadequate, and the metabolic patterns of various agents are not fully elucidated, complicating precise dose individualization.

Given the high TB incidence and mortality, alongside complex treatment in hemodialysis patients with ESRD, the present study retrospectively analyzed clinical data from 90 such cases, systematically evaluated the efficacy and safety of different treatment regimens in patients, and proposed the following hypotheses: Personalized dose adjustment and administration timing can significantly improve the cure rate of patients, reduce mortality, and improve the quality of life of patients. By exploring the PK characteristics of anti-TB drugs in ESRD patients receiving hemodialysis, we can better understand the process of drug absorption, distribution, metabolism, and excretion in the body, provide scientific guidance for reasonable adjustment of drug dose and dosing regimen, and avoid poor efficacy or increased adverse reactions caused by improper drug dose. The present study is expected to provide a more optimized plan for the clinical treatment of ESRD patients with TB, fill part of the gap in PK research and clinical treatment in this field, and play an important role in improving the overall treatment level of ESRD patients with TB.

## Methods

### Study design

**Figure 1.**
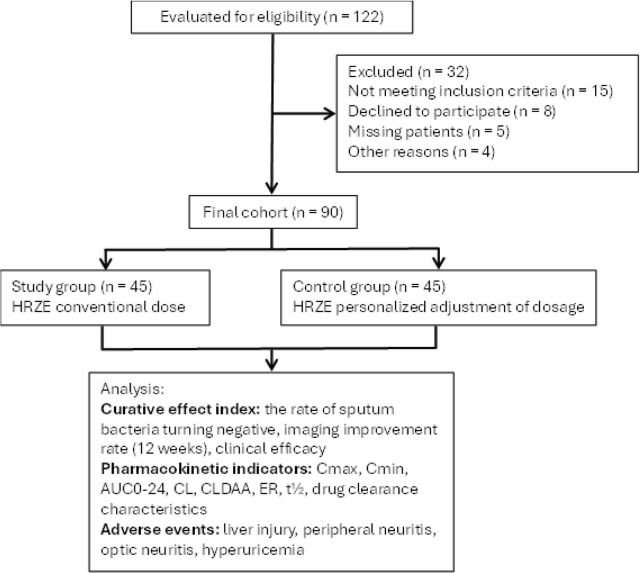
Design flowchart. CL, clearance rate.

This retrospective controlled study initially screened 122 patients with ESRD and active TB on maintenance hemodialysis, treated between January 2020 and January 2024, via electronic medical record review. After exclusions—15 not meeting inclusion criteria, 8 declining participation, 5 lost to follow-up, and 4 for other reasons—90 patients were included. These participants were allocated into a control group (n = 45), receiving conventional anti-TB dosing, and a study group (n = 45), receiving individualized dose and timing adjustments. Medication compliance was evaluated through pill counting and direct observation of treatment on dialysis days. Patients with poor compliance (missing 10% of the dose) were excluded from the analysis. In addition, in accordance with the national TB control policy, it is recommended to conduct contact investigations on all family members and close caregivers of the enrolled patients. However, the results of these investigations are not part of the data collection for this clinical efficacy study, and the results of these investigations are not analyzed in the present study. The design operation process is shown in **Figure 1**.

The study was approved by the Ethics Committee of Baoding People’s Hospital (approval number 2023-01). All participants provided written informed consent; in emergency situations, their legal representatives or guardians were permitted to provide consent on their behalf. All data involved in the study were anonymized to ensure the privacy and confidentiality of the participants.

### Inclusion and exclusion criteria

Inclusion criteria were as follows: (1) the age ranged from 18 to 85 years, both sexes; (2) meeting the clinical diagnostic criteria of ESRD [18]; (3) no history of TB or signs of active TB before dialysis; (4) duration of dialysis was ≥3 months; (5) bacteriologically or pathologically confirmed active TB (pulmonary tuberculosis/extrapulmonary TB); (6) ESRD patients who underwent maintenance hemodialysis (MHD); and (7) awareness of the purpose and content of the present study and signed the relevant consent form.

Exclusion criteria were as follows: (1) co-infection with human immunodeficiency virus (HIV), solid organ transplantation, or active malignancy; (2) severe liver dysfunction (Child-Pugh class C) or baseline alanine aminotransferase (ALT) >3 times the upper limit of normal; (3) pregnant or lactating women; (4) pretreatment sputum smear revealing multidrug-resistant TB; (5) received continuous renal replacement therapy or peritoneal dialysis during the study; and (6) allergic to the study drugs.

### Treatment methods

#### Hemodialysis protocol

All patients underwent hemodialysis 3 times per week (Monday, Wednesday, and Friday, 13:00–17:00) using a high-flux dialyzer (Fx80, Fresenius, Germany). Each session lasted ≥4 h, with a target weekly Kt/V of ≥2.0 [18]. Standard bicarbonate dialysate was used with a constant temperature of 36.5°C. Blood flow and dialysate flow rates were maintained at 200–250 mL/min and 500 mL/min, respectively. Anticoagulation was managed per unit protocol, and vital signs were monitored routinely throughout the procedure.

#### Anti-TB treatment

All patients received a 12-week HRZE (isoniazid, rifampicin, pyrazinamide, ethambutol)-based regimen. The treatment protocols for the 2 groups were as follows:

Control group—basic anti-TB treatment according to WHO TB treatment guidelines [19]: (1) rifampicin (Shenyang Hongqi; H21021905): 10 mg/kg, 1 time/d, 30 m after the end of dialysis; (2) isoniazid (Shanghai Shangyao Xinyi; H31020495): 5 mg/kg, once a day, 30 m after the end of dialysis; (3) pyrazinamide (Shenyang Hongqi; H21022354): 25 mg/kg, once a day, 30 m after the end of dialysis; and (4) ethambutol (Hangzhou Minsheng; H33021602): 15 mg/kg, once a day, 4–6 h before dialysis.

Study group—according to the “Chinese Expert Consensus on the management of adult CKD with TB” [20], the following adjustments were made. (1) Rifampicin: the daily dose of rifampicin was adjusted to 10 mg/kg (body weight <50 kg, 450 mg/d; body weight ≥50 kg, 600 mg/d). On the non-dialysis days, the normal dose of 10 mg/kg was given, 30 m after the end of dialysis; (2) Isoniazid: dose adjustment to 5 mg/kg (maximum 300 mg), administered 30 m after the end of dialysis on non-dialysis days, and dialysis day additional 50% dose (2.5 mg/kg) 4 h after dialysis; (3) Pyrazinamide: dose adjusted to 25–35 mg/kg (maximum 2000 mg), 3 times a week, 30 m after the end of dialysis; and (4) Ethambutol: dose adjusted to 15–25 mg/kg (maximum 1200 mg) 3 times a week, 30 m after the end of dialysis.

#### Adjunctive medication

All patients must take vitamin B6 orally (25–50 mg/day) on non-dialysis days.

### Observation indicators

(1) Baseline data of patients were collected before treatment, including demographic characteristics, type of primary renal disease, dialysis parameters, TB characteristics, laboratory indicators, immune indicators, comorbidities, and medication history.
(2) Sputum negative conversion rate: morning sputum samples were collected by Ziehl-Neelsen acid-fast staining on 3 consecutive days at weeks 4, 8, and 12 after the start of treatment, and double-blind reading by 2 experienced laboratory technicians, with 2 consecutive negative smear results at least 24 h apart.
(3) Imaging evaluation: after 12 weeks of treatment, the cavity closure rate, infiltration focus absorption rate, and pleural effusion absorption rate were recorded by imaging examination.
(4) Clinical efficacy: After the treatment is completed, the therapeutic effect is judged based on the patient’s clinical symptoms, imaging examinations, and etiological examination results [21]. It is divided into 4 grades: cured, markedly effective, effective, and ineffective. Cure: Clinical symptoms completely disappear, imaging examinations show complete absorption of the lesion, and etiological tests are negative. Marked effect: Clinical symptoms are significantly improved, imaging examinations show that most of the lesions have been absorbed, and etiological tests are negative. Effective: Clinical symptoms have improved, imaging examinations show partial absorption of the lesion, and etiological tests may be positive. Ineffective: There is no improvement or aggravation of clinical symptoms. Imaging examinations show no change or expansion of the lesion. Etiological tests are positive. The effective rate of treatment = (number of cured cases + number of markedly effective cases + number of effective cases)/total number of cases × 100%.
(5) PK index detection: Blood samples (3–5 mL) were collected into EDTA-containing vacuum tubes before dialysis, during dialysis (2 h, 4 h), after dialysis (0.5 h, 4 h), and before the next dialysis. The collected samples were immediately centrifuged at 3000 rpm for 10 m at 4°C. The obtained plasma was ali-quoted into cryogenic vials and stored at −80°C until analysis. All samples were transferred on dry ice to the central laboratory for batch processing. The drug concentration was determined by liquid chromatography-tandem mass spectrometry (LC-MS/MS). Monitoring instruments and analytical conditions: An ANASTAR liquid chromatography workstation equipped with an SSI500 detector (SSI Company, USA) was used. A Kromasil C18 chromatographic column (5 µm, 250 mm × 46 mm, Dalian Institute of Physical Chemistry, Chinese Academy of Sciences) was employed. Drug reference substances were provided by the National Institutes for Food and Drug Control. Non-compartmental analysis (NCA) was performed using WinNonlin 8.1. An ACQUITY UPLC HSS T3 column (2.1 × 100 mm, 1.8 µm) was used to detect rifampicin/ethambutol (detection limit: 0.1 µg/mL), isoniazid (detection limit: 0.05 µg/mL), and pyrazinamide (detection limit: 0.2 µg/mL). PK parameters and calculations: The following PK parameters were assessed. C_max_ (peak concentration) is the highest plasma drug concentration observed after administration. C_min_ (trough concentration) is the drug concentration measured immediately before the next administration. AUC_0-24_ (area under the 0-24 h drug-time curve) was calculated using the linear trapezoidal method: AUC_0-24_ = Σ [(C_i_ + C_i+1_)/2 × (t_i+1_ - t_i_)]. Clearance (CL) represents the volume of plasma cleared of drug per unit time, calculated as CL = dose / AUC_0–∞_ (after single administration). CLDAA (dialysis clearance rate) was determined by collecting blood samples from the dialyzer inlet (arterial end, CA) and outlet (venous end, CV), as well as dialysate effluent samples (CDiA), with simultaneous measurement of dialy-sate flow rate (QD) and blood flow rate (QB): CLDAA = QB × [(CA - CV)/CA]. ER (extraction rate) is the proportion of drug removed during a single pass through the dialyzer: ER = (CA - CV)/CA. t^½^ (half-life) is the time required for drug concentration to decrease by half, calculated as t^½^ = 0.693/Ke, where Ke is the terminal elimination rate constant. PK sampling protocol: PK sampling was conducted after at least two weeks of consistent anti-TB therapy to ensure that drug concentrations were at steady state.
(6) Adverse reactions: Record all adverse events during the treatment period, including liver injury, peripheral neuritis, optic neuritis, and hyperuricemia.

### Sample size calculation

The sample size was determined *a priori* using G*Power software. An effect size of 0.63, a significance level (α) of 0.05 for a 2-tailed test, and a statistical power (1-β) of 0.8 were specified. The analysis indicated that 40 participants per group (80 total) were required. Ultimately, 90 patients were enrolled, exceeding the minimum sample size and thereby satisfying statistical requirements for the study design.

### Statistical analysis

All statistical evaluations were executed with SPSS 25.0, whereas flow diagrams were generated via Lucidchart. The Shapiro–Wilk test assessed distributional normality, and Levene’s procedure appraised variance homogeneity. Continuous baseline characteristics are summarized as mean ± SD; inter-group contrasts were examined with independent-samples *t*-tests. Categorical indices are presented as counts (percentages); group differences were analyzed using either the χ^2^ test or Fisher’s exact approach, as appropriate. The intergroup differences in the negative conversion rate of sputum bacteria, the improvement rate of imaging, and the clinical efficacy were correlated as odds ratio (OR) and 95% confidence interval (CI) (95% CI). Primary PK indices and the influence of hemodialysis on plasma levels were reported as mean ± SD. Repeated measurements of the main PK indicators at multiple time points (before dialysis, during dialysis, and after dialysis) were conducted using repeated measures analysis of variance (ANOVA). Frequencies of adverse events were contrasted with the *χ^2^* statistic. Every inferential test was 2-tailed, and statistical significance was set at *P* < 0.05.

## Result

### Comparison of baseline data

A total of 90 patients with ESRD complicated with TB and receiving maintenance hemodialysis were included in the present study. They were divided into the control group and the study group according to the administration regimen, with 45 cases in each group. The comparison results of baseline characteristics show that demographic characteristics (age, proportion of males, and BMI), types of primary kidney diseases (diabetic nephropathy, hypertensive nephropathy, chronic glomerulonephritis, and polycystic kidney disease), dialysis parameters (dialysis age, residual urine volume, dialysis method, and Kt/V value), TB features (proportion of pulmonary TB, extrapulmonary TB, cavitary pulmonary TB, positive sputum smear grade, and TB symptoms score), laboratory indicators (hemoglobin, serum creatinine, urea nitrogen, albumin, hs-CRP, blood calcium, blood phosphorus, and iPTH), immune indicators (CD4 + T cell count, CD8 + T cell count, CD4 + /CD8 + ratio, IL-6). There were no significant differences in all baseline indicators such as comorbidities (diabetes, coronary heart disease, and hypertension) and drug use history (previous anti-TB treatment history and immunosuppressant use history) (*P* > 0.05), indicating that the 2 groups of patients were comparable in basic characteristics before treatment, as shown in **Table 1**.

**Table 1.** Comparison of baseline characteristics between the 2 groups (*x̄* ± *s*)

**Indicators**	**Control group (n = 45)**	**Study group (n = 45)**	**Statistical (*χ^2^/t)***	***P***
**Demographic characteristics**				
Age (years)	59.1 ± 10.2	58.3 ± 9.7	*t* = 0.37	0.707
Proportion of males	60.0% (27)	62.2% (28)	*χ^2^* = 0.08	0.765
BMI (kg/m^2^)	21.9 ± 2.8	22.4 ± 3.1	*t* = −0.79	0.430
**Primary kidney disease type**				
Diabetic nephropathy	46.7% (21)	44.4% (20)	*χ^2^* = 0.05	0.823
Hypertensive nephropathy	26.7% (12)	28.9% (13)	*χ^2^* = 0.06	0.806
Chronic glomerulonephritis	22.2% (10)	20.0% (9)	*χ^2^* = 0.07	0.792
Polycystic kidney	4.4% (2)	6.7% (3)	FET	1
**Dialysis parameters**				
Dialysis age (months)	38.3 ± 20.1	38.5 ± 11.1	*t* = 1.11	0.269
Residual urine volume (mL/24 h)	145 ± 72	152 ± 68	*t* = −0.84	0.403
Dialysis method (HDF:HD)	26:19	28:17	*χ^2^* = 0.18	0.669
Kt/V value	1.49 ± 0.23	1.52 ± 0.21	*t* = −0.62	0.531
**Characteristics of** TB				
TB	84.4% (38)	86.7% (39)	*χ^2^* = 0.09	0.761
Extrapulmonary TB	15.6% (7)	13.3% (6)	*χ^2^* = 0.09	0.761
The proportion of cavitary pulmonary TB	73.3% (33)	75.6% (34)	*χ^2^* = 0.06	0.806
Classification of positive sputum smears (1 + /2 + /3+)	16/17/6	18/15/6	*χ^2^* = 0.32	0.852
TB symptom score (0–10 points)	6.5 ± 1.3	6.2 ± 1.5	*t* = 1.00	0.320
**Laboratory indicators**				
Hemoglobin (g/L)	96.8 ± 11.7	98.5 ± 12.3	*t* = −0.66	0.508
Serum creatinine (µmol/L)	792.5 ± 142.8	768.2 ± 156.4	*t* = 0.76	0.449
Urea nitrogen (mmol/L)	29.1 ± 7.2	28.3 ± 6.7	*t* = 0.54	0.591
Albumin (g/L)	34.7 ± 3.9	35.2 ± 4.1	*t* = −0.58	0.559
hs-CRP (mg/L)	20.1 ± 6.8	18.6 ± 7.3	*t* = 1.09	0.275
Blood calcium (mmol/L)	2.08 ± 0.29	2.12 ± 0.31	*t* = −0.60	0.546
Blood phosphorus (mmol/L)	1.92 ± 0.38	1.86 ± 0.42	*t* = 0.70	0.485
iPTH (pg/mL)	372.6 ± 166.2	348.5 ± 152.7	*t* = 1.10	0.273
**Indicators of immunity**				
CD4 + T cell count (cells/µL)	298 ± 102	312 ± 108	*t* = −0.75	0.454
CD8 + T cell count (cells/µL)	703 ± 142	685 ± 156	*t* = 0.56	0.573
CD4 + /CD8 + ratio	0.42 ± 0.14	0.46 ± 0.15	*t* = −1.28	0.203
IL-6 (pg/mL)	26.1 ± 9.5	24.3 ± 8.7	*t* = 1.17	0.242
**Comorbidity**				
Diabetes	55.6% (25)	51.1% (23)	*χ^2^* = 0.18	0.669
Coronary heart disease	26.7% (12)	24.4% (11)	*χ^2^* = 0.07	0.792
Hypertension	71.1% (32)	75.6% (34)	*χ^2^* = 0.24	0.624
**History of drug use**				
Previous history of anti-TB treatment	13.3% (6)	15.6% (7)	*χ^2^* = 0.10	0.752
History of immunosuppressive therapy	11.1% (5)	8.9% (4)	FET	0.732

TB, tuberculosis. FET: Fisher’s exact test. HDF: hemodiafiltration; HD: hemodialysis.

### Analysis of efficacy index results

#### Dynamic changes in the rate of sputum bacteria turning negative

After 4 weeks of treatment, the sputum negative conversion rate in the study group reached 62.2%, significantly exceeding the 35.6% observed in the control group (*P* = 0.011). By week 8, the conversion rate further increased to 86.7% in the study group, compared to 60.0% in the control group (*P* = 0.004). After 12 weeks of treatment, the negative conversion rate of the study group was 93.3% and that of the control group was 73.3%, as shown in **Table 2**.

**Table 2.** The negative conversion rate of sputum bacteria in the 2 groups changed dynamically (%, n/N)

**Time point**	**Control group (n = 45)**	**Study group (n = 45)**	**OR**	**95% CI**	***P***
4 weeks of treatment	35.6% (16/45)	62.2% (28/45)	3.27	1.52–7.05	0.011
8 weeks of treatment	60.0% (27/45)	86.7% (39/45)	4.33	1.71–10.98	0.004
12 weeks of treatment	73.3% (33/45)	93.3% (42/45)	5.09	1.43–18.11	0.011

CI, confidence interval; OD, odds ratio.

#### Imaging improvement rate (12 weeks)

After 12 weeks of treatment, the cavity closure rate in the study group was significantly higher than that in the control group (*P* = 0.009), and the cavity closure rate of 76.5% in the study group was close to the internationally recognized threshold for treatment success (>75%), significantly reducing the risk of subsequent transmission, while the control group was still under treatment (<50%). The absorption rate of infiltrative foci was 82.2% in the study group, significantly higher than that in the controls (*P* = 0.034). Similarly, pleural effusion absorption reached 88.9% in the study group compared to 68.9% in the control group, as shown in **Table 3**.

**Table 3.** Rates of radiographic improvement at week 12 in both groups (%, n/N)

**Parameters**	**Control group (n = 45)**	**Study group (n = 45)**	**OR**	**95% CI**	***P***
Cavity closure rate	48.9% (22/45)	76.5% (34/45)	3.23	1.32–7.93	0.009
Absorption rate of the infiltrated lesion	62.2% (28/45)	82.2% (37/45)	2.81	1.06–7.43	0.034
Absorption rate of pleural effusion	68.9% (31/45)	88.9% (40/45)	3.61	1.17–11.12	0.020

CI, confidence interval; OD, odds ratio.

#### Comparison of clinical efficacy

After treatment, 36 cases were cured, 6 cases were markedly effective, 2 cases were effective, and 1 case was ineffective in the study group. In the control group, 18 cases were cured, 10 cases were markedly effective, 8 cases were effective, and 9 cases were ineffective. The overall efficacy of the study group was significantly better than that of the control group (*P* < 0.05) (**Table 4**).

**Table 4.** Comparison of clinical efficacy between the 2 groups (*x̄* ± *s*)

**Groups**	**n**	**Curing**	**Markedly effective**	**Effective**	**Ineffective**	***χ* ^2^**	***P***
Control group	45	18 (40.0%)	10 (22.2%)	8 (17.8%)	9 (20.0%)	12.94	<0.001
Study group	45	36 (80.0%)	6 (13.3%)	2 (4.4%)	1 (2.2%)		

### Analysis of PK index results

#### Comparison of key PK parameters

The PK parameters of the anti-TB drugs are summarized in **Table 5**. Compared to the control group, the study group demonstrated significantly higher C_max_, C_min_, and AUC_0–24_ for all 4 anti-TB drugs (all *P* < 0.05). CL was significantly lower in the study group for isoniazid, pyrazinamide, and ethambutol (*P* < 0.05), while no significant difference was observed for rifampicin (*P* > 0.05). The t^½^ was significantly longer in the study group for all drugs (*P* < 0.05).

**Table 5.** Comparison of key PK parameters between the 2 groups (*x̄* ± *s*)

**Parameter**	**H**	**R**	**Z**	**E**
C_max_ (µg/mL)				
Control group (n = 45)	5.6 ± 0.9	12.5 ± 1.8	25.7 ± 4.3	5.4 ± 0.8
Study group (n = 45)	8.9 ± 1.5**	16.8 ± 2.4**	38.5 ± 5.6**	8.2 ± 1.1**
C_min_ (µg/mL)				
Control group (n = 45)	0.6 ± 0.2	1.2 ± 0.3	9.3 ± 1.7	0.4 ± 0.1
Study group (n = 45)	1.5 ± 0.4**	2.8 ± 0.6**	12.8 ± 2.1**	0.9 ± 0.2**
AUC_0–24_ (µg_**·**_h/mL)				
Control group (n = 45)	28.9 ± 5.2	56.7 ± 8.9	287.5 ± 42.6	24.7 ± 3.5
Study group (n = 45)	45.6 ± 6.7**	85.6 ± 12.3**	412.8 ± 58.3**	35.2 ± 4.8**
t^½^ (h)				
Control group (n = 45)	4.3 ± 0.9	4.8 ± 0.7	12.4 ± 1.9	11.7 ± 1.6
Study group (n = 45)	5.8 ± 1.1**	5.2 ± 0.8*	16.8 ± 2.5**	12.5 ± 1.8*
CL (L/h)				
Control group (n = 45)	34.6 ± 5.2	10.6 ± 1.8	4.3 ± 0.7	24.2 ± 3.1
Study group (n = 45)	22.0 ± 3.5**	7.0 ± 1.2**	3.0 ± 0.5**	17.0 ± 2.3**

* *P* < 0.05.

** *P* < 0.01 indicates a statistically significant difference compared with the control group.

#### Effect of dialysis on drug concentration

The present study analyzed the clearance properties of different anti-TB drugs by high-flux dialysis. The results showed CL_DAA_, the dialysis clearance rate of ethambutol, was the highest, reaching 172.8 ± 18.7 mL/min, followed by isoniazid, 142.5 ± 15.3 mL/min. Dialysis clearance of rifampicin and pyrazinamide was relatively low, at 65.4 ± 7.2 mL/min and 58.7 ± 6.5 mL/min, respectively. ER, the extraction rate of ethambutol, was the highest (0.72 ± 0.08), indicating that the dialyzer had the highest removal efficiency. Isoniazid (0.59 ± 0.06), the extraction yields of rifampicin and pyrazinamide, was lower, at 0.27 ± 0.03 and 0.24 ± 0.03, respectively. The half-life of all anti-TB drugs on dialysis day was significantly shortened (*P* < 0.01) (**Table 6**).

**Table 6.** Effect of dialysis on clearance of major antituberculosis drugs (*x̄* ± *s*)

**Parameter**	**H**	**R**	**Z**	**E**
CL_DAA_	142.5 ± 15.3	65.4 ± 7.2	58.7 ± 6.5	172.8 ± 18.7
ER	0.59 ± 0.06	0.27 ± 0.03	0.24 ± 0.03	0.72 ± 0.08
Non-dialysis day t^½^ (h)	5.8 ± 1.1	5.2 ± 0.8	16.8 ± 2.5	12.5 ± 1.8
Dialysis day t^½^ (h)	3.2 ± 0.6^#^	2.8 ± 0.5^#^	9.4 ± 1.3^#^	6.5 ± 1.0^#^

# *P* < 0.01 indicates a statistically significant difference compared with non-dialysis days.

#### Analysis of safety index results

The incidence of liver injury, optic neuritis, and hyperuricemia in the study group was significantly lower than that in the control group (*P* < 0.05). There was no significant difference in peripheral neuritis between the study group and the control group (*P* = 1.000). The total adverse reaction rate in the study group was 24.4%, which was significantly lower than 62.2% in the control group (*P* < 0.001), indicating that the dose adjustment regimen in the study group was safer than the conventional anti-TB regimen (**Table 7**).

**Table 7.** Incidence of adverse reactions (n (%))

**Adverse events**	**Control group (n = 45)**	**Study group (n = 45)**	***χ* ^2^**	***P***
Liver injury	22.2% (10/45)	6.7% (3/45)	4.68	0.031
Optic neuritis	15.6% (7/45)	0% (0/45)	7.50	0.006
Hyperuricemia	31.1% (14/45)	13.3% (6/45)	4.18	0.041
Peripheral neuropathy	8.9% (4/45)	8.9% (4/45)	0.00	1.000
Total adverse reactions	62.2% (28/45)	24.4% (11/45)	13.31	<0.001

## Discussion

Due to the impaired immune system function, patients with ESRD have a significantly higher risk of TB than the general population. The process of HD further affects the body’s physiological state and complicates anti-TB treatment. The present study focused on ESRD patients with TB receiving maintenance hemodialysis and assessed the clinical effectiveness and PK properties of a personalized anti-TB dosing strategy, thereby informing treatment optimization.

The study group demonstrated significantly higher rates of sputum conversion, radiographic improvement at 12 weeks, and overall treatment effectiveness compared to the conventional-dose control group (*P* < 0.05). At the 4-week mark, the sputum conversion rate reached 62.2% in the study group, markedly surpassing the control group’s (35.6%, OR = 3.27, 95% CI: 1.52–7.05, *P* = 0.011). This indicates that the probability of the research group achieving sputum transformation in the fourth week was >3 times higher than that of the control group and approaching the response range typically seen in immunocompetent TB patients (65%–70%). This result was even higher than the data of TB patients reported by Kim et al. [22], and the negative conversion rate was about 30.8%–52.7% in 4 weeks. After 8 weeks of treatment, the sputum-negative conversion rate of the study group was 86.7%. The rate of sputum bacteria turning negative after 2 months of anti-TB treatment was highly consistent with that reported by Zhang et al. [23]. Moreover, at 12 weeks of treatment, the negative conversion rate of the study group reached 93.3%, while that of the control group was only 73.3% (OR = 5.09, 95% CI: 1.43–18.11, *P* = 0.011). At the end of the 12-week period, the probability of successful sputum transformation for patients receiving individualized treatment regimens was approximately 5 times higher. This gap highlights the sustained antibacterial effect of the individualized regimen. Imaging evaluation at 12 weeks revealed a cavity closure rate of 76.5% in the study group, significantly exceeding the 48.9% rate observed in the control group (OR = 3.23, 95% CI: 1.32–7.93, *P* = 0.009), which was equivalent to a 3.2 times higher probability of lumen closure in the study group. This result is close to the study by Zhai et al. [24] on ESRD pulmonary TB (with a cavity closure rate of approximately 50% in the standard protocol) but markedly surpassed the findings observed in their research. The research group significantly improved the overall treatment by adjusting the dosage and administration timing. This comprehensive therapeutic advantage has been confirmed in multiple similar studies. The retrospective study results of Saito et al. [25] suggest that adjusting the dosage of TB drugs for CKD patients according to the guidelines has a similar therapeutic effect to that of non-CKD patients. Moreover, results from this investigation demonstrate that prompt and effective anti-TB therapy markedly alleviates clinical symptoms in ESRD patients with TB co-infection. The research results of Caixia et al. [26] suggest that for maintenance hemodialysis patients with tuberculous peritonitis, active anti-TB treatment can achieve better results. In the present study, the clinical symptoms of the patients in the research group improved more significantly under individualized treatment, further affirming the beneficial impact of optimized treatment regimens on patient quality of life.

The changes in PK parameters are the focus of the present study. In the present study, the dose adjustment protocol successfully improved the drug exposure level, and the C_max_ and AUC_0–24_ of all 4 anti-TB drugs in the study group were significantly higher than those in the control group (*P* < 0.05). The significant clinical efficacy observed in the study group can be attributed to the attainment of the expected PK parameters of the anti-TB drug. For rifampicin, the AUC_0–24_/MIC ratio is considered to be the key indicator for judging the success of treatment, and it is generally believed that the target value should be more than 175 and the C_max_ should be between 5 and 20 µg/mL. Previous studies have shown that the C_max_ of isoniazid is greater than 3–5 µg/mL, which is essential for optimal bactericidal effect and to prevent the development of resistance. Pyrazinamide AUC_0–24_ >300 µg·h/mL was associated with favorable treatment outcomes. Ethambutol concentrations usually have a C_max_ of 5–10 µg/mL at 2–4 h after dose, and our personalized treatment regimen successfully achieved this goal [27]. In terms of C_max_, the peak concentrations of isoniazid, rifampicin, pyrazinamide, and ethambutol in the research group increased by 59%, 34%, 50%, and 52%, respectively. This increase is comparable to the study described in Sileshi et al. [28] on CKD patients (where C_max_ increased by 30%–55% after dose adjustment), but it significantly improves the antibacterial effect. In terms of drug exposure (AUC_0–24_), all 4 core drugs in the research group achieved an increase of over 40%. Consistent with the prospective study conclusion of Chigutsa et al. [29], the present study pointed out that the AUC/MIC ratio is the most crucial PK/PD parameter for predicting the treatment outcome of TB in ESRD patients. Especially in the application of pyrazinamide, our individualized protocol enabled the AUC_0–24_ to reach 412.8 µg·h/mL, which was significantly higher than the 287.5 µg**·**h/mL of the control group. This optimization has special value because previous literature, such as Putra et al. [30] and Thumamo et al. [31], generally hold that patients with ESRD should avoid using pyrazinamide, fearing that the accumulation of its metabolites may lead to hyperuricemia [30, 32]. However, in the study group, significant reductions in the CL values of isoniazid, pyrazinamide, and ethambutol were observed. This was the result of optimizing the dosing regimen rather than changes in the patients’ intrinsic metabolic or excretory capabilities. Since CL is calculated by dose/AUC, the higher AUC obtained through our personalized adjustment of the dose directly leads to a lower calculated CL value. This indicates more effective drug utilization rather than altering the fundamental clearance pathways. In addition, CL_DAA_ of ethambutol in the present study was 172.8 ± 18.7 mL/min, and ER was 0.72, which means that conventional dialysis can remove approximately 68.5% of the drug. This phenomenon was also observed in the study by Mondal et al. [33], who reported that the clearance rate of ethambutol during 4-h dialysis reached 60%–75%. The PK characteristics of rifampicin show particularity in the present study. There was no significant change in its CL, which is consistent with the mechanism by which rifampicin is mainly metabolized by the liver and gallbladder and is less dependent on the kidneys for excretion. This finding is consistent with the recommendation of the Expert Consensus on the Treatment of CKD Complicated with TB (2022 Edition) [34], that is, rifampicin does not need to adjust the dose according to GFR in ESRD patients, but the concentration supplementation on dialysis day needs to be paid attention to. Compared with previous studies, this research further clarified the impact of dialysis on the half-life of drugs. The half-lives of all anti-TB drugs on dialysis day were significantly shortened (*P* < 0.01), which was consistent with other literature reports. As Gu et al. [35] pointed out, the hemodialysis process accelerates drug clearance, leading to a shortened half-life. Therefore, the timing and dosage of administration need to be adjusted according to the dialysis duration. In the present study, the research group maintained the effective duration of the drugs in the body and ensured the continuous antibacterial effect by rationally supplementing the drugs after dialysis.

Safety represents a critical dimension in treatment evaluation. The incidence of adverse reactions was substantially lower in the study group compared to that in the control group (*P* < 0.01), which is an important advantage of individualized dose-adjusted anti-TB regimens. This advantage was particularly prominent in liver injury (6.7% vs 22.2%, *P* = 0.031) and optic neuritis (0% vs 15.6%, *P* = 0.006). It is worth noting that although the exposure to pyrazinamide in the study group was significantly increased, the incidence of hyperuricemia was actually lower than that in the control group (13.3% vs 31.1%). This is not quite consistent with previous studies; however, a deeper analysis reveals that the research group adjusted the administration time of pyrazinamide to after dialysis and combined it with adequate dialysis to clear uric acid precursors, so that the peak concentration of the drug was synchronized with the peak clearance of dialysis, thereby avoiding the continuous accumulation of metabolites. This strategy provides a new idea for the safe application of pyrazinamide in patients with ESRD. The incidence of peripheral neuritis in both groups was 8.9%, confirming that the preventive use of vitamin B6 was effective for both groups. This finding supports the latest expert consensus recommendation that all ESRD patients receiving isoniazid should routinely supplement vitamin B6 (25–50 mg/d), regardless of whether they receive dose adjustment or not. Therefore, the dose-adjusting treatment plan in the present study is relatively safe and will not increase the risk of medication.

## Conclusion

For ESRD patients undergoing hemodialysis combined with TB, adjusting the dosage and administration timing of anti-TB drugs based on PK characteristics can significantly improve clinical efficacy, enhance the PK parameters of drugs in the body, reduce the incidence of adverse reactions, and provide a safe and effective individualized management path for anti-TB treatment. Although the present study achieved meaningful results, there are still certain limitations: First, the sample size is limited, which may affect the detection of rare adverse reactions; Second, the present study did not track the long-term prognosis of the patients. Subsequent research can focus on indicators such as the long-term survival rate and recurrence rate of the patients. Third, no subgroup analysis was conducted, making it impossible to assess the differences in therapeutic effects among patients with different characteristics. Future studies can extend the follow-up period to evaluate long-term efficacy, expand the sample size to verify security, and conduct subgroup analysis to identify dominant populations and to explore individualized treatment plans.
